# Intra-instar larval cannibalism in *Anopheles gambiae* (*s.s*.) and *Anopheles stephensi* (Diptera: Culicidae)

**DOI:** 10.1186/s13071-016-1850-5

**Published:** 2016-11-02

**Authors:** Daniele Porretta, Valentina Mastrantonio, Graziano Crasta, Romeo Bellini, Francesco Comandatore, Paolo Rossi, Guido Favia, Claudio Bandi, Sandra Urbanelli

**Affiliations:** 1 Department of Environmental Biology, Sapienza University of Rome, Rome, Italy; 2 Department of Mathematics, Sapienza University of Rome, Rome, Italy; 3 Agriculture Environment Centre “G. Nicoli”, Crevalcore, Italy; 4 Department of Veterinary Science and Public Health, University of Milan, Milan, Italy; 5 Department of Biology and Biotechnology, University of Pavia, Pavia, Italy; 6 School of Bioscience and Veterinary Medicine, University of Camerino, Camerino, Italy

**Keywords:** Intraspecific predation, Larval behavior, Malaria vectors, Mosquitoes

## Abstract

**Background:**

Cannibalism has been observed in a wide range of animal taxa and its importance in persistence and stability of populations has been documented. In anopheline malaria vectors the inter-instar cannibalism between fourth- and first-instar larvae (L4-L1) has been shown in several species, while intra-instar cannibalism remains poorly investigated. In this study we tested the occurrence of intra-instar cannibalism within larvae of second-, third- and fourth-instar (L2, L3 and L4) of *Anopheles gambiae* (*s.s*.) and *An. stephensi*. Experiments were set up under laboratory conditions and the effects of larval density, duration of the contact period among larvae and the presence of an older larva (i.e. a potential cannibal of bigger size) on cannibalism rate were analysed. Cannibalism was assessed by computing the number of missing larvae after 24 and 48 h from the beginning of the experiments and further documented by records with a GoPro videocamera.

**Results:**

Intra-instar cannibalism was observed in all larval instars of both species with higher frequency in *An. gambiae* (*s.s*.) than in *An. stephensi*. In both species the total number of cannibalistic events increased from 0–24 to 0–48 h. The density affected the cannibalism rate, but its effect was related to the larval instar and to the presence of older larvae. Interestingly, the lower cannibalism rate between L4 larvae was observed at the highest density and the cannibalism rate between L3 larvae decreased when one L4 was added.

**Conclusions:**

The present study provides experimental evidence of intra-instar cannibalism in the malaria vectors *An. gambiae* (*s.s*.) and *An. stephensi* and highlights the possible occurrence of complex interactions between all larval instars potentially present in the breeding sites. We hypothesize that the high density and the presence of a potential cannibal of bigger size could affect the readiness to attack conspecifics, resulting into low risk larval behavior and lower cannibalism rate. The understanding of cannibalistic behavior and the factors affecting it is of utmost importance for malaria vectors, as nutrition during larval development can strongly affect the fitness of adult female mosquitoes and ultimately their vector ability.

**Electronic supplementary material:**

The online version of this article (doi:10.1186/s13071-016-1850-5) contains supplementary material, which is available to authorized users.

## Background

Cannibalism (i.e. the killing and eating of entire or parts of conspecific individuals) has had an awkward history and has initially been interpreted as an uncommon accident or an artifact of experimental conditions [1]. In the last decades, the mounting data that have documented its occurrence have changed this view and now it is recognized that cannibalism occurs in a wide range of animal taxa [1–7].

In last few decades several studies addressed the adaptive significance of cannibalism as well as its ecological effects. At a population level, cannibalism can significantly affect persistence and stability of populations [1–6]. Cannibalism can indeed drastically reduce population size and maintain it below the carrying capacity of the environment, contributing to self-regulation of population size. In the ladybird *Harmonia axyridis* cannibalism can lead to up to 50 % of the newly ecloded individuals’ mortality as well as among grasshoppers, locusts, and Mormon crickets, can be a major cause of mortality in the field [8–12]. Furthermore, cannibalism could increase the resilience of a population to environmental stressors as cannibal individuals and survivors to cannibalism are likely the more vigorous individuals and those that can better respond to environmental stress [5, 13]. Finally, cannibalism has been suggested to increase the ability of population propagules to colonize and persist in new stressful environments by affecting the dispersal, nutritional ability and development time of individuals [3, 14, 15]. In the red flour beetle *Tribolium castaneum*, cannibalism has been suggested to facilitate colonization of marginal environments and in larvae of the harlequin ladybird *Harmonia axyridis*, cannibalism was significantly greater in invasive than in native or laboratory populations [14, 15].

At an individual level, cannibalism has been suggested to have several benefits, the most important being nutritional [2, 16]. The additional food gained has been shown to increase development rate, survival and fertility of cannibals in several arthropod species [1, 3, 7]. In the pierid *Ascia monuste* cannibal caterpillars showed higher survival rate and weight than noncannibal ones [16]. Likewise, in *Drosophila melanogaster* larvae, it was shown that an exclusively cannibalistic diet was sufficient for normal development from eggs to fertile adults [17]. In this species enhanced propensity toward cannibalism was also found after 118 generations of rearing under malnutrition conditions, supporting its adaptive role [17].

On the other hand, cannibalistic behavior can be also harmful or have fitness costs. By eating potential partners, cannibals can reduce potential mates or contract pathogens and parasites from their victims [1, 3, 6, 13, 18]. Furthermore, trying to eat the victim, cannibals can be injured. In this context, attacking smaller individuals can reduce the risk to be injured and this could account for the frequent occurrence of size-dependent cannibalism [1, 3, 7]. However, size-independent cannibalism is not unusual and among insects it has been observed in larvae of *Toxorhynchites* mosquitoes, in the moth *Carmenta hematica,* the chrysomelid beetle *Labidomera clivicollis* [1, 6] as well as at high population density in the desert locust *Schistocerca gregaria*, where cannibalistic interactions have been suggested to drive the coordinated mass migration processes of juvenile stages [11]. Cannibalism is therefore a context-dependent process, in which environmental factors, such as the quality and quantity of diet, population density, availability and behavior of the potential victims can significantly affect the costs and benefits associated to cannibalistic behavior [1, 3, 6, 14].

In mosquitoes, larval cannibalism has been documented in several species and genera [19–21]. Larval mosquitoes develop in temporary aquatic environments of very different dimensions and origin (small pools, cattle hoof prints, tyre tracks of tractors, man-made holes and containers, river edges, ponds, marshes) [19, 22]. The dynamics and the rapidly changing conditions of these environments can lead to extreme competition between larvae occupying a breeding site [19–21, 23, 24]. Inter- and intra-specific competition between larval instars has been documented in several species as well as it has been shown that competition can significantly affect larval survival or development [19–24]. Under these conditions, cannibalism removing potential competitors and furnishing food intake can be a selectively favored and widespread behavior [1, 3].

In anopheline malaria vectors cannibalism between fourth- and first-instar larvae (inter-instar) has been observed in several species [20, 21], including the Asian vector *Anopheles stephensi* [25] and the African vectors *An. gambiae* (*s.s*.) and *An. arabiensis* [21]. The nutritional benefits of cannibalism can affect larval nutrition, survival and, ultimately, adult fitness and vector ability [26–28]. Well-nourished larvae result indeed in larger adult females which also exhibit a large fitness and vector ability than females emerging from malnourished larvae [27–30]. In the malaria vector *An. stephensi* it has been shown that an increase of larval food quantity significantly affected adult life-history traits related to fitness such as survival, length of gonotrophic cycle, and number of emerging adults [27]. Likewise, it has been shown that *An. gambiae* (*s.s*.) and *An. stephensi* adult females emerged from larvae reared under nutritionally high diets contained more metabolic reserves and higher prevalence and intensity of *Plasmodium yoelii nigeriensis* than females emerged from nutritionally low larvae [26]. The understanding of cannibalistic behavior, the factors underlying it and its consequence on larval fitness can therefore be of particular interest for malaria vectors.

Contrary to inter-instar cannibalism, experimental studies focusing on cannibalism between larvae of the same instar (intra-instar) remain scarce, although it could occur in nature, because in larval breeding sites several cohabiting larvae of the same instar are commonly observed, as females lay several eggs at one site and avoid ovipositing where older larvae are present [31].

In this paper we aimed to investigate the possible occurrence of intra-instar cannibalism in the malaria vectors *An. gambiae* (*s.s*.) and *An. stephensi*. We explicitly tested the occurrence of cannibalistic behavior within larvae of second-, third-, and fourth-instar and investigated the possible influence of larval density, duration of the contact period among larvae, and the presence of an older instar larva (i.e. a potential cannibal of bigger size) on cannibalism rate.

Density has been previously shown to affect inter-instar cannibalism in both species. In *An. gambiae* (*s.s*.) cannibalism between fourth- and first- instar larvae increased with density of the first-instar larvae [21], showing a positive relationship. Likewise, in *An. stephensi*, cannibalism experiments between fourth- and first-instar larvae showed that nearly all the first-instar larvae were consumed during the 48 h of the experiment and that the number of consumed larvae increased with the time of contact between them (from zero to 24 and 48 h), and with the density of both first-instar and fourth-instar larvae [25]. To explain these observations, it has been suggested that cannibalism could be a consequence of crowding, that would lead to the violation of the minimum individual distance between individuals, or an accidental consequence of normal feeding activities [3]. In *An. stephensi*, it has been suggested that the observed cannibalism between fourth- and first-instar larvae could arise from circular currents created by the filtering action of mouth brushes of the fourth-instar larvae sweeping up first-instar larvae into the mouth parts [25]. Cannibalism rate has been therefore suggested to be the final product of random contacts between cannibal and victims [25]. Under this hypothesis, in our experiments, we would expect that cannibalism will increase with larval density and/or time of contact between larvae of *An. gambiae* (*s.s*.) and *An. stephensi*. Likewise, we would expect increased cannibalism when an older larva is added as a consequence of the encounters between larvae of the same instar as well as of different instars.

## Methods

### Mosquitoes

The mosquito larvae used in this study were obtained from the *Anopheles gambiae* (*s.s*.) and *Anopheles stephensi* Liston colonies maintained in the insectary of the University of Camerino, Italy. Rearing conditions were 29 °C temperature, 85–90 % relative humidity, 12:12 L-D photoperiod. Adults were maintained in 25 × 25 × 25 cm cages and daily fed with 5 % sucrose solution. Larvae were maintained in spring water and daily fed with fish food (0.85 mg/larva) (Tetra, Melle, Germany). Experiments were performed using eggs hatched at intervals of two days to provide larvae of the desired instar from second- to fourth-instar (L2, L3 and L4). Newly hatched first-instar larvae were maintained as described above until they were used for the experiments.

### Experimental setup

Experiments were set up in wells of plastic ice-cube trays (2 × 4 × 3 cm each well) filled with spring water. The size and shape of the experimental wells and the water volume used were designed to allow both vertical and horizontal movements of larvae. All experiments were performed under the same temperature, humidity and light/dark conditions of the reared colonies. Mosquito larvae are omnivores and the lack of food is an unrealistic condition in nature [19]. We therefore provided food to the larvae during the experiments according to the rearing conditions described above (0.85 mg/larva of fish food daily). We tested the occurrence of intra-instar cannibalism using L2, L3 and L4 larvae and assessed the influence of larval density, duration of the contact period among larvae and the presence of an older larva in the experimental well (i.e. of a potential cannibal of bigger size) on cannibalism rate.

Two, five and ten larvae of the same instar (L2, L3 and L4) were placed into the experimental wells filled with 10 ml of spring water, leading to a density of 0.2, 0.5 and 1.0 larvae/ml, respectively [14]. To test the effect of the contact time among larvae on cannibalism, the number of missing larvae in each well was computed after 24 and 48 h from the beginning of the experiments. Further treatments were performed to test the effect on intra-instar cannibalism of an older larva. As described above, two, five and ten L2 and L3 larvae were placed into experimental wells, then one L3 was placed into (L2-L2) wells, one L4 into (L2-L2) wells and one L4 into the (L3-L3) wells, which resulted in (L2-L2)L3, (L2-L2)L4 and (L3-L3)L4 treatments. Missing larvae were computed after 24 and 48 h. In all treatments food was supplied at the beginning of the experiment and after 24 h. For each experimental condition ten replicates were performed. The replicates with L4 larvae where pupation occurred were discarded. Controls were also performed using dead larvae to test for possible larval decomposition and disappearance. Two, five and ten dead L2, L3 and L4 larvae (killed at -80 °C for 5 min) were placed into the wells which were maintained under the same experimental conditions as before. Missing larvae were counted after 48 h.

To further document the possible occurrence of cannibalism, under each treatment conditions, 15 min movies were recorded using a GoPro camera positioned above the wells. All experiments started in the morning (about 11:00 am). *Anopheles gambiae* (*s.s*.) treatments were recorded in the afternoon (from about 2:00 pm) with the following order: (L2-L2) (0.2, 0.5, 1.0 larvae/ml); (L2-L2)L3 (0.2, 0.5, 1.0 larvae/ml, respectively); (L2-L2)L4 (0.2, 0.5, 1.0 larvae/ml); (L3-L3); (L3-L3)L4 (0.2, 0.5, 1.0 larvae/ml); (L4-L4) (0.2, 0.5, 1.0 larvae/ml). In the afternoon of the second day, *Anopheles stephensi* treatments were recorded following the same order described above. A total of 9 h of records were performed. Movies were edited using the GoPro STUDIO software.

### Data analyses

The rate of cannibalism in each experimental condition was computed as n_tot_/[(N-1) × 10], where n_tot_ is the number of missing larvae in the ten wells of each experimental condition; N is the total number of larvae placed in each well, [(N-1) is therefore the maximum number of possible cannibalism events, as at least one larva, i.e. the cannibal, is expected to remain in each well)]; and 10 is the number of wells for each experimental condition. Pairwise comparisons between cannibalism rate observed under the experimental conditions were performed using Chi-square test. The effect of the experimental factors and their combination on the cannibalism rate was investigated by logistic regression, using cannibalism as a binary response variable. The experimental factors used were: larval instar (L2, L3, L4); density of the larvae of the same instar; time (i.e. the duration of contact between larvae, 24 and 48 h); presence of the older larva (encoded as: absence = 0; presence of L3 = 1; presence of L4 = 2). After explorative analyses, for each species we designed six nested models using the above factors and their combinations. In the Model 1 we tested the effect of all above factors: (instar) + (density) + (time) + (older larva). The Model 2 tested the effect of time by removing this factor from the Model 1: (instar) + (density) + (older larva). The Models 3–5 tested the combined effect of some factors: Model 3, [(instar × density) + (time) + (older larva)]; Model 4, [(density) + (time) + (instar × older larva)]; Model 5, [(instar × density) + (time) + (instar × older larva)]. Finally, the Model 6 tested the effect of time when the other factors were combined as in Model 5: [(instar × density) + (instar × older larva)].

We tested the fit of the most complex model of the set (i.e. the Model 5) with the observed data using the Hosmer and Lemeshow goodness-of-fit test [32]. Then the models were compared by their Akaike information criterion (AIC) value and pairwise Chi-square tests were used to test statistically significant differences. The model with the lowest AIC is the “best” model among all models specified for the dataset used. To further assess the effect of the experimental factors on the cannibalism rate, ANOVA analysis was performed for the Model 5, that is the global model and that best fits the observed data (see Results section). All analyses were performed separately for *Anopheles gambiae* (*s.s*.) and *An. stephensi* using the data collected at 48 h. All analyses were performed using the software R 3.0.2 [33].

## Results

### Cannibalism occurrence

No dead larvae were observed after 24 h, while a mortality of 1.8 and 1.1 % was observed after 48 h in *An. gambiae* (*s.s*.) and *An. stephensi*, respectively. In both species we found larval disappearance under all experimental conditions. Two replicates were discarded in *An. gambiae* (*s.s*.) and two in *An. stephensi* as a consequence of L4 pupation. Different cannibalism rates were observed for the two species as, by considering the overall datasets, more larvae disappeared in *An. gambiae* (*s.s*.) (190/919 = 20.7 %) than in *An. stephensi* (51/925 = 5.5 %) (*χ* ^2^ = 71.999, *df* = 1, *P* = 0.0001) during the 48 h. In all control tests with dead larvae, no larval disappearance was observed, showing no larval decomposition during the 48 h.

To further document the possible occurrence of cannibalism, we performed 15 min movies for each experimental test using a GoPro camera positioned above the wells for a total of 9 h. No cannibalism was observed in any movie with the exception of one in *An. gambiae* (*s.s*.), where cannibalism was observed in (L3-L3)L4 treatment at 1.0 larvae/ml, where one L4 ate one L3 (see the movie in Additional file 1: Movie).Additional file 1: Movie. The movie shows a cannibalism event in *Anopheles gambiae* (*s.s*.), where one L4 larva ate one L3. (MOV 102129 kb)


### Factors affecting cannibalism rate

The *Anopheles gambiae* (*s.s*.) and *An. stephensi* datasets were analysed separately. Although the overall cannibalism rate was significantly lower in *An. stephensi* than in *An. gambiae* (*s.s*.), similar patterns were observed in the two species.

**Fig. 1:**
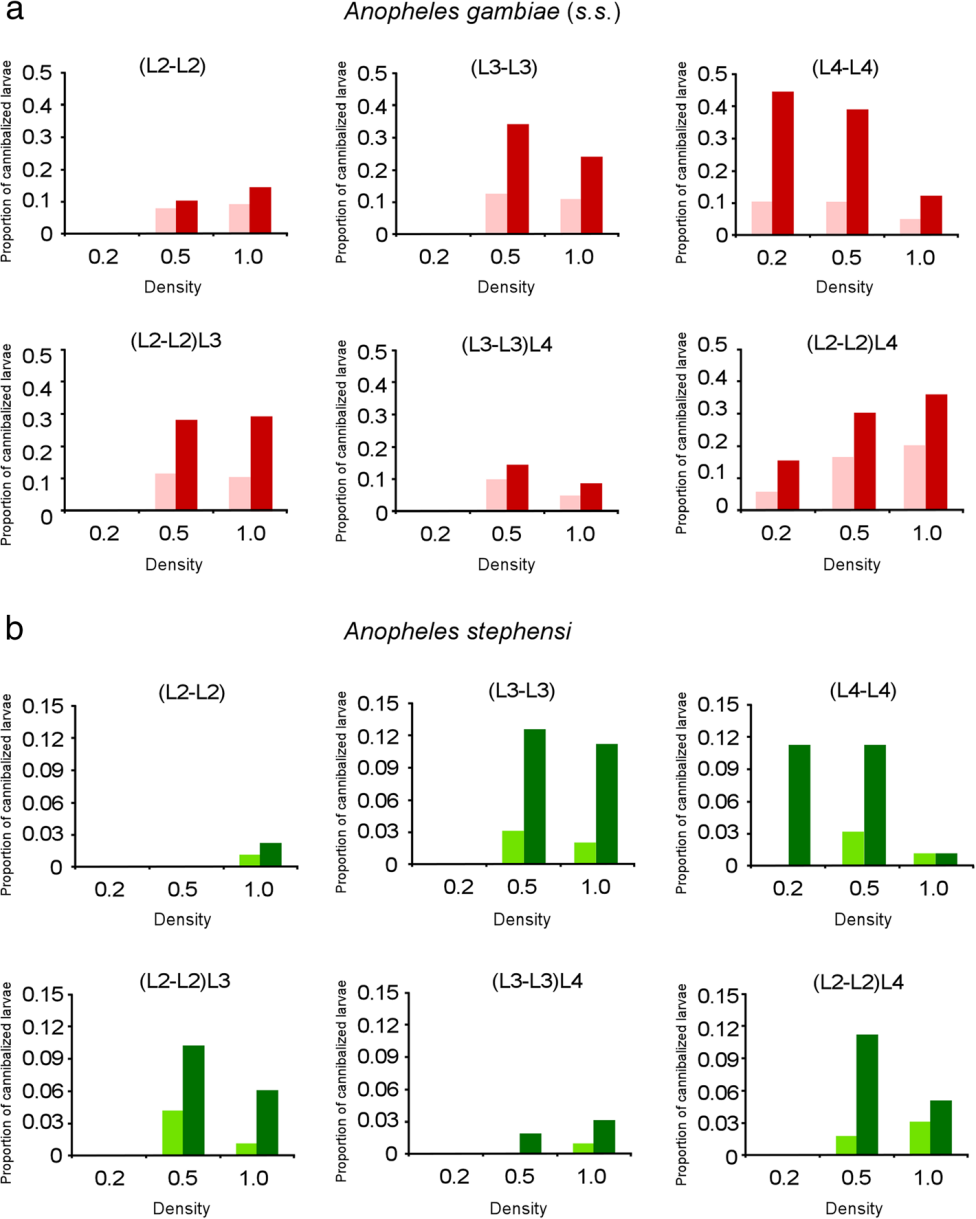
Cannibalism rates in *Anopheles gambiae* (*s.s*.) and *Anopheles stephensi*. Proportion of missing larvae observed in the experimental treatments after 24 and 48 h is shown. **a** Cannibalism observed in *An. gambiae* (*s.s*.) after 24 h (*pink*) and 48 h (*red*). **b** Cannibalism observed in *An. stephensi* after 24 h (*light green*) and 48 h (*dark green*). Density refers to the larvae of the same stage in each well (larvae/ml)

In *An. gambiae* (*s.s*.), the Hosmer and Lemeshow goodness-of-fit test [32] showed that the Model 5 (i.e. the most complex model of the set) [(instar × density) + (time) + (instar × older larva)], fitted the observed data well (*χ* ^2^ = 3.14, *df* = 8, *P* = 0.93). Model 5 was also the model that explained the observed data better than the other models tested by logistic regression analysis (AIC value = 698.29, all pairwise *χ* ^2^ tests *P* < 0.05) (Table 1). Under this model, the cannibalism rate is significantly affected by the contact time between larvae. The Models 2 and 6, indeed, where the factor time was excluded, had significantly higher AIC value than Model 5 (pairwise *χ* ^2^ tests, *P* < 0.05). By considering all experimental conditions, the total number of cannibalism events increased from 0–24 to 0–48 h (87/919 = 9.5 % at 24 h, 190/919 = 20.7 % at 48 h; *χ* ^2^ = 33.963, *df* = 1, *P* = 0.0001) (Fig. 1). Cannibalism events between 0–24 h (87 larvae disappeared) and 24–48 h (103 larvae disappeared) showed no significant differences (*χ* ^2^ = 3.83, *df* = 1, *P* = 0.054), although the *P*-value observed was at the limit of significance. Among the other factors analyzed, the Model 5 supports a major role of the combination of larval instar and density, suggesting that density affected the cannibalism rate, but in different ways in L2, L3 and L4 larvae. In (L2-L2) and (L3-L3) treatments, no cannibalism was observed at 0.2 larvae/ml and no significant differences were found between the cannibalism rate at 0.5 and 1.0 larvae/ml (Additional file 2: Table S1). In (L4-L4) treatments, no significant differences were found between cannibalism rate under 0.2 and 0.5 larvae/ml densities, while significantly lower cannibalism rate was found under the higher density, 1.0 larvae/ml (pairwise *χ* ^2^ tests *P* < 0.05) (Additional file 2: Table S1; Fig. 1).

**Table 1:** Logistic regression analyses. The effects of experimental factors and their interactions on cannibalism rate in *Anopheles gambiae* (*s.s*.) and *An. stephensi* were tested separately for each species

Model		Null variance	*df*	Residual variance	*df*	AIC
*An. gambiae* (*s.s.*)						
Model 1	(stage) + (density) + (time) + (older larva)	489.85	355	433.54	349	741.24
Model 2	(stage) + (density) + (older larva)	489.85	355	478.33	350	784.02
Model 3	[(stage × density) + (time) + (older larva)]	489.85	355	406.91	347	718.61
Model 4	[(density) + (time) + (stage × older larva)]	489.85	355	395.91	348	705.61
Model 5	[(stage × density) + (time) + (stage × older larva)]	489.85	355	371.31	346	685.01
Model 6	[(stage × density) + (stage × older larva)]	489.85	355	417.67	347	729.04
*An. stephensi*						
Model 1	(stage) + (density) + (time) + (older larva)	208.68	355	185.67	349	304.63
Model 2	(stage) + (density) + (older larva)	208.68	355	206.06	350	323.02
Model 3	[(stage × density) + (time) + (older larva)]	208.68	355	179.65	347	302.61
Model 4	[(density) + (time) + (stage × older larva)]	208.68	355	169.58	348	290.53
Model 5	[(stage × density) + (time) + (stage × older larva)]	208.68	355	163.32	346	288.28
Model 6	[(stage × density) + (stage × older larva)]	208.68	355	183.95	347	306.91

Under the Model 5, the combination of larval instar and presence of an older larva in the experimental well (i.e. of a potential bigger cannibal) significantly affected the cannibalism rate as well. Instar-specific influence of older larvae was also supported by pairwise *χ* ^2^ tests: in (L2-L2)L3 and (L2-L2)L4 treatments cannibalism rate increased significantly with respect to (L2-L2) treatments under both 0.5 and 1.0 larvae/ml. On the contrary, in (L3-L3)L4 treatments lower cannibalism rates were observed than in (L3-L3) treatments under both 0.5 and 1.0 larvae/ml conditions (pairwise *χ* ^2^ tests, *P* < 0.05) (Additional file 2: Table S1).

In *An. stephensi*, as in *An. gambiae* (*s.s*.), the Model 5 (i.e. the most complex model of the set), fitted the observed data well (Hosmer and Lemeshow goodness-of-fit test, *χ* ^2^ = 1.46, *df* = 8, *P* = 0.99) and showed the lowest AIC value (all pairwise *χ* ^2^ tests, *P* < 0.05). Under this model, the cannibalism rate is significantly affected by the contact time between larvae. Indeed, the Models 2 and 6, where the factor time was excluded, showed the highest AIC values (Table 1), and by considering all experimental conditions the number of disappeared larvae significantly increased from 0–24 to 0–48 h (14/925 = 1.5 % at 24 h, 51/925 = 5.5 % at 48 h, *χ* ^2^ = 20.354, *df* = 1, *P* = 0.0001) (Additional file 2: Table S1). Cannibalism at 0–24 h was significantly lower than at 24–48 h (14 and 37 disappeared larvae, respectively) (*χ* ^2^ = 11.033, *df* = 1, *P* = 0.0009).

Under the Model 5, larval density and the presence of an older larva in combination with larval instar, significantly affected the cannibalism rate as well. In (L2-L2) treatments no cannibalism was observed at 0.2 and 0.5 larvae/ml and in (L3-L3) treatments at 0.2 larvae/ml. In (L3-L3) treatments pairwise comparisons showed no significant differences between the cannibalism rate after 48 h at 0.5 and 1.0 larvae/ml (pairwise *χ* ^2^ tests *P* > 0.05). In (L4-L4) treatments, no significant differences were found in cannibalism rate between 0.2 and 0.5 larvae/ml density, while significantly lower cannibalism was found at 1.0 larvae/ml than at 0.2 and 0.5 larvae/ml (pairwise *χ* ^2^ tests *P* < 0.05) (Additional file 2: Table S1). With respect to the influence of the presence of older larvae, cannibalism rate significantly increased between (L2-L2) and (L2-L2)L3 and (L2-L2)L4 treatments at 0.5 larvae/ml. On the contrary, in (L3-L3)L4 treatments lower cannibalism rates were observed than in (L3-L3) treatments at both 0.5 and 1.0 larvae/ml (all pairwise *χ* ^2^ tests, *P* < 0.05) (Additional file 2: Table S1).

Concordantly with the logistic regression analyses, ANOVA showed that the factor time and the combination of the factors (instar × density) and (instar × older larva) significantly affected the cannibalism observed in both *An. gambiae* (*s.s.*) and *An. stephensi* (Additional file 3: Table S2).

## Discussion

The inter-instar cannibalism between L4 and L1 larvae has been observed in several mosquitoes species, including anopheline vectors, while intra-instar cannibalism remains poorly investigated, although some clues can be found in literature [25, 34]. For example, in *Anopheles stephensi*, in cannibalism experiments between L4 and L1 larvae, nearly all the first-instar larvae were consumed during the 48 h of the experiment, however, in the experimental cups few fourth-instar larvae disappeared as well, which would suggest the possible occurrence of cannibalism among them [25]. In this study, we tested the occurrence of intra-instar cannibalism in L2, L3 and L4 larvae in *An. gambiae* (*s.s*.) and in *An. stephensi* and found evidences for it in all of them. In all experimental tests (L2-L2), (L3-L3) and (L4-L4), the disappearance of larvae was indeed observed both after 24 and 48 h. At least three considerations support that these larvae disappeared as a consequence of cannibalistic behavior: (i) in control tests with dead larvae, no disappearance of larvae over the 48 h of the experiment was observed, allowing us to exclude larval decomposition; (ii) scavenger behavior was not observed under our experimental conditions and dead larvae were found also in wells where no cannibalism was observed; (iii) the movies allowed us to directly observe cannibalism.

Different cannibalism rates were observed between *An. gambiae* (*s.s*.) and *An. stephensi*, with lower cannibalism in *An. stephensi.* This result is not surprising as it is recognized that cannibalism frequency can significantly differ among taxa, including closely related species or populations from different geographical areas [3–6, 14]. Further studies using other *An. gambiae* (*s.s*.) and *An. stephensi* strains as well as natural populations across their geographical range could allow to assess how general are the differences here observed between the two species and the factors underlying them.

The experimental approach used also allowed us to analyze the possible factors affecting the cannibalism rate observed within the two species. A positive relationship between the contact time and the total number of cannibalism events has been observed in inter-instar experiments on mosquito species, such as in *An. stephensi* [25], as well as in intra-instar experiments such as in *Toxorhynchites rutilus* [35] and *Armigeres subalbatus* [36]. Here we found that in both *An. gambiae* (*s.s*.) and *An. stephensi*, the duration of the contact between larvae significantly affected the cannibalism rate and, consistently with the above studies, the total number of cannibalism events increased from 24 to 48 h in all experimental treatments (Fig. 1). These observations likely rely on fact that the increase of the contact time can increase the probability for cannibal and victims to bump into each other, or, the frequency of cannibal-victim interactions. However, significant differences were observed between 0–24 and 0–48 intervals in *An. stephensi* and marginal significance was observed in *An. gambie* (*s.s.*) which could suggest that cannibalism is likely more than a probabilistic process and a behavioral component could play a role as well. Our results about the effects of density on cannibalism rate further support the involvement of behavioural components.

In both *An. gambiae* (*s.s*.) and *An. stephensi* we found that larval density affected the cannibalism rate, but its effect was related to larval instar and the presence of older larvae (Table 1). In both (L2-L2) and (L3-L3) treatments cannibalism increased from 0.2 larvae/ml density to the higher densities. However, no differences were observed between 0.5 and 1.0 larvae/ml. In (L4-L4) treatment, no differences were observed between 0.2 and 0.5 larvae/ml, while the lowest cannibalism rate was observed at the highest density analyzed. These results can hardly be explained by an effect of higher probability of encounter and led us to hypothesize the occurrence of behavioral factors in cannibals and/or victims.

Cannibalism is the result of a cost-benefits balance [3]. The relative sizes of cannibals and victims can therefore be crucial in cannibalistic interaction. Attacking smaller individuals reduces the risk to be injured or killed and enhances the probability of successfully gain a prey (i.e. reduces the risk and maximizes the benefits) [1, 3]. When larvae of different size co-occur, bigger larvae would reduce the risks and maximize the benefits by attacking smaller individuals. On the contrary, when larvae of similar size co-occur, the risk to attack each other is higher, as each larva can be injured by the potential victim as well as it is exposed to the attacks by other larvae. We can therefore hypothesize that under high density, particularly in (L4-L4) treatment, the cannibal is more exposed to the risk to be in turn attacked and/or not enough free space is available to launch safe attacks. Both factors could increase the risk of injury, resulting in lower cannibalism rate. This cost-benefits hypothesis could also support the results observed in the experimental tests where larger larvae were added. In the (L3-L3)L4 treatment, the cannibalism rate decreased, regardless of the density (Fig. 1, Additional file 2: Table S1), which is consistent with the hypothesis that in the presence of an additional risk (i.e. the presence of a L4 larva) the L3 larvae reduced the attacks towards each other. Finally, in the (L2-L2)L3 and (L2-L2)L4 treatments the cannibalism rate increased with respect to (L2-L2) treatments, showing likely an effect of both intra- and inter-instar cannibalism (although we cannot assess the relative frequency and/or if L2-L2 cannibalism is inhibited).

Studies on antipredator behavior of mosquito larvae have shown that they are able to gauge predation risk from chemical and mechanical cues as well as displaying active antipredator responses such as escapes during attacks, avoidance behaviors and reduced mobility to elude predators [19, 37]. Our small experimental arenas can mimic conditions for mosquitoes developing in small breeding sites. Furthermore, mosquito larvae show aggregation behavior in the breeding sites that could reduce the effects of the size differences between small and larger developing sites [19]. However, we could expect that the topography of developing sites could affect the cannibalistic rate by affecting the cost/benefit balance of cannibalistic behavior. For example, we could expect that in experimental cells with more space available than in our experimental conditions, the number of attacks will increase as the risk of injuries could decrease in more complex and bigger spaces. On the other hand, successful attacks could be reduced when refugia are present or the escape chances are higher. Interestingly, in the mosquito *Toxorhynchites amboinensis*, it has been shown that cannibalism rate was significantly affected by the container shape rather than by the container volume per se [38]. Future experiments manipulating the size, shape and complexity of the experimental cell will help to investigate habitat scaling effects on cannibalism rate as well as to test the hypotheses that we proposed to explain our observed results.

## Conclusions

In this study we provided for the first time experimental evidences of intra-instar cannibalism in the malaria vectors *An. gambiae* (*s.s*.) and *An. stephensi* and analysed some factors affecting cannibalism rate. Our results emphasize the context-dependent nature of cannibalism in these species and, adding to the previous evidences of L4-L1 cannibalism, highlight the possible occurrence of more complex interactions between all larval instars potentially present in the breeding sites.

## Additional files

Additional file 2: Table S1. Pairwise comparisons between cannibalism rates observed after 48 h in *Anopheles gambiae* (*s.s*.) and *Anopheles stephensi* in the experimental treatments using L2, L3 and L4 larvae. Density refers to larvae of the same instar in each well (larvae/ml). *P*-values < 0.05 are shown in bold. (DOC 68 kb)
Additional file 3: Table S2. ANOVA analysis performed for the global model (Model 5) based on cannibalism data at 48 h in *Anopheles gambiae* (*s.s.*) and *An. stephensi*. (DOC 37 kb)

## Abbreviations

L2: Second-instar larvae
L3: Third-instar larvae
L4: Fourth-instar larvae
