# Supplementary material for: Intra-instar larval cannibalism in Anopheles gambiae (s.s.) and Anopheles stephensi (Diptera: Culicidae)
Source: Parasit Vectors. 2016 Nov 2;9:566. doi: 10.1186/s13071-016-1850-5 (PMC5094080; doi:10.1186/s13071-016-1850-5)
Supplement: Additional file 2: Table S1. — Pairwise comparisons between cannibalism rates observed after 48 h in Anopheles gambiae (s.s.) and Anopheles stephensi in the experimental treatments using L2, L3 and L4 larvae. Density refers to larvae of the same instar in each well (larvae/ml). P-values < 0.05 are shown in bold. (DOC 68 kb) [file 13071_2016_1850_MOESM2_ESM.doc]

**Additional file 2: Table S1. Pairwise comparisons between cannibalism rates observed after 48 hours in *Anopheles gambiae* (*s.s*.) and *Anopheles stephensi* in the experimental treatments using L2, L3 and L4 larvae. Larval instar comparisons are also described in the text. Density refers to larvae of the same instar in each well (larvae/ml). *P*-values < 0.05 are shown in bold**

| **Pair-wise *χ*^2^ tests** | |  | ***Anopheles gambiae* (s.s.)** | | |  | ***Anopheles stephensi*** | | |
| --- | --- | --- | --- | --- | --- | --- | --- | --- | --- |
| **Larval-Stage**  **comparisons** | **Density**  **(larvae/ml)** |  | ***χ*^2^** | ***df*** | ***P*-value** |  | ***χ*^2^** | ***df*** | ***P*-value** |
|  |  |  |  |  |  |  |  |  |  |
| (L2-L2) *vs* (L2-L2) | 0.2 *vs* 0.5 |  | 1.087 | 1 | 0.2971 |  | - | - | - |
|  |  |  |  |  |  |  |  |  |  |
| (L2-L2) *vs* (L2-L2) | 0.2 *vs* 1.0 |  | 1.660 | 1 | 0.1976 |  | 0.227 | 1 | 0.6339 |
|  |  |  |  |  |  |  |  |  |  |
| (L2-L2) *vs* (L2-L2) | 0.5 *vs* 1.0 |  | 0.481 | 1 | 0.4879 |  | 0.227 | 1 | 0.6339 |
|  |  |  |  |  |  |  |  |  |  |
|  |  |  |  |  |  |  |  |  |  |
| (L3-L3) *vs* (L3-L3) | 0.2 *vs* 0.5 |  | 4.392 | 1 | **0.0361** |  | 1.389 | 1 | 0.2386 |
|  |  |  |  |  |  |  |  |  |  |
| (L3-L3) *vs* (L3-L3) | 0.2 *vs* 1.0 |  | 3.134 | 1 | 0.0767 |  | 1.235 | 1 | 0.2665 |
|  |  |  |  |  |  |  |  |  |  |
| (L3-L3) *vs* (L3-L3) | 0.5 *vs* 1.0 |  | 0.913 | 1 | 0.3392 |  | 0.052 | 1 | 0.819 |
|  |  |  |  |  |  |  |  |  |  |
|  |  |  |  |  |  |  |  |  |  |
| (L4-L4) *vs* (L4-L4) | 0.2 *vs* 0.5 |  | 0.149 | 1 | 0.6993 |  | 0.0 | 1 | 1.0 |
|  |  |  |  |  |  |  |  |  |  |
| (L4-L4) *vs* (L4-L4) | 0.2 *vs* 1.0 |  | 6.608 | 1 | **0.0102** |  | 4.134 | 1 | **0.0420** |
|  |  |  |  |  |  |  |  |  |  |
| (L4-L4) *vs* (L4-L4) | 0.5 *vs* 1.0 |  | 11.059 | 1 | **0.0009** |  | 6.748 | 1 | **0.0094** |
|  |  |  |  |  |  |  |  |  |  |
|  |  |  |  |  |  |  |  |  |  |
| (L2-L2) *vs* (L2-L2)L3 | 0.5 *vs* 0.5 |  | 4.5 | 1 | **0.00339** |  | 4.235 | 1 | **0.0396** |
|  |  |  |  |  |  |  |  |  |  |
| (L2-L2) *vs* (L2-L2)L4 | 0.5 *vs* 0.5 |  | 5.337 | 1 | **0.0209** |  | 4.235 | 1 | **0.0396** |
|  |  |  |  |  |  |  |  |  |  |
| (L2-L2) *vs* (L2-L2)L3 | 1.0 *vs* 1.0 |  | 5.828 | 1 | **0.0158** |  | 1.676 | 1 | 0.1954 |
|  |  |  |  |  |  |  |  |  |  |
| (L2-L2) *vs* (L2-L2)L4 | 1.0 vs 1.0 |  | 10.696 | 1 | **0.0011** |  | 1.030 | 1 | 0.310 |
|  |  |  |  |  |  |  |  |  |  |
|  |  |  |  |  |  |  |  |  |  |
| (L3-L3) *vs* (L3-L3)L4 | 0.5 *vs* 0.5 |  | 4.4 | 1 | **0.0359** |  | 3.938 | 1 | **0.0472** |
|  |  |  |  |  |  |  |  |  |  |
| (L3-L3) *vs* (L3-L3)L4 | 1.0 *vs* 1.0 |  | 9.634 | 1 | **0.0019** |  | 4.889 | 1 | **0.027** |
