# Supplementary material for: Intra-instar larval cannibalism in Anopheles gambiae (s.s.) and Anopheles stephensi (Diptera: Culicidae)
Source: Parasit Vectors. 2016 Nov 2;9:566. doi: 10.1186/s13071-016-1850-5 (PMC5094080; doi:10.1186/s13071-016-1850-5)
Supplement: Additional file 3: Table S2. — ANOVA analysis performed for the global model (Model 5) based on cannibalism data at 48 h in Anopheles gambiae (s.s.) and An. stephensi. (DOC 37 kb) [file 13071_2016_1850_MOESM3_ESM.doc]

**Additional file 3: Table S2.** ANOVA analysis performed for the global model (Model 5) based on cannibalism data at 48 h in *Anopheles gambiae* (*s.s.*) and *An. stephensi*.

|  | Df | Deviance Resid. | Df Resid. | Dev | Pr(>Chi) |
| --- | --- | --- | --- | --- | --- |
| *Anopheles gambiae* (*s.s.*) |  |  |  |  |  |
| Null |  |  | 355 | 489.85 |  |
| Instar | 2 | 8.601 | 353 | 481.25 | 0.01356* |
| Density | 1 | 2.721 | 352 | 478.53 | 0.09903 |
| Time | 1 | 44.782 | 351 | 433.75 | 2.203e-11*** |
| Older larva | 2 | 0.210 | 349 | 433.54 | 0.90045 |
| Instar × Density | 2 | 26.627 | 347 | 406.91 | 1.652e-6*** |
| Instar × Older larva | 1 | 35.602 | 346 | 371.31 | 2.421e-9 *** |
|  |  |  |  |  |  |
| *Anopheles stephensi* |  |  |  |  |  |
| Null |  |  | 355 | 208.68 |  |
| Instar | 2 | 0.4132 | 353 | 208.27 | 0.81333 |
| Density | 1 | 0.0133 | 352 | 208.25 | 0.90822 |
| Time | 1 | 20.3656 | 351 | 187.89 | 6.397e-06*** |
| Older larva | 2 | 2.2165 | 349 | 185.67 | 0.33014 |
| Instar × Density | 2 | 6.0199 | 347 | 179.65 | 0.04929* |
| Instar × Older larva | 1 | 16.3282 | 346 | 163.32 | 5.327e-5*** |

**P* < 0.05 ****P* < 0.001
